# Process Parameters for FFF 3D-Printed Conductors for Applications in Sensors

**DOI:** 10.3390/s20164542

**Published:** 2020-08-13

**Authors:** Tibor Barši Palmić, Janko Slavič, Miha Boltežar

**Affiliations:** Faculty of Mechanical Engineering, University of Ljubljana, Aškerčeva 6, 1000 Ljubljana, Slovenia; tibor.barsi@fs.uni-lj.si (T.B.P.); miha.boltezar@fs.uni-lj.si (M.B.)

**Keywords:** additive manufacturing, material extrusion, fused-filament fabrication, polymer nanocomposite, process parameters, conductive filament

## Abstract

With recent developments in additive manufacturing (AM), new possibilities for fabricating smart structures have emerged. Recently, single-process fused-filament fabrication (FFF) sensors for dynamic mechanical quantities have been presented. Sensors measuring dynamic mechanical quantities, like strain, force, and acceleration, typically require conductive filaments with a relatively high electrical resistivity. For fully embedded sensors in single-process FFF dynamic structures, the connecting electrical wires also need to be printed. In contrast to the sensors, the connecting electrical wires have to have a relatively low resistivity, which is limited by the availability of highly conductive FFF materials and FFF process conditions. This study looks at the Electrifi filament for applications in printed electrical conductors. The effect of the printing-process parameters on the electrical performance is thoroughly investigated (six parameters, >40 parameter values, >200 conductive samples) to find the highest conductivity of the printed conductors. In addition, conductor embedding and post-printing heating of the conductive material are researched. The experimental results helped us to understand the mechanisms of the conductive network’s formation and its degradation. With the insight gained, the optimal printing strategy resulted in a resistivity that was approx. 40% lower than the nominal value of the filament. With a new insight into the electrical behavior of the conductive material, process optimizations and new design strategies can be implemented for the single-process FFF of functional smart structures.

## 1. Introduction

Combining multi-material fused-filament fabrication (FFF) and functional composite materials enables the fabrication of an object with embedded functional elements (sensorics [1], actuation [2], heating [3], and energy storage [4], etc.) in a single-step printing process.

FFF 3D printing is based on the deposition of the melted filament feedstock onto the build surface. Sequentially deposited traces of melted material in horizontal layers form the fused printed structure. Filament FFF materials must exhibit a melting point in the working temperature range of the FFF 3D printer and a suitable viscosity so the material can be melted and extruded through the nozzle. Filaments can be manufactured with the addition of various filler particles with functional properties that enable the fabrication of functional structures.

Conductivity is achieved with various conductive fillers, based on conductive carbon nanomaterials and metals that are mixed with a thermoplastic matrix to ensure the printability of the composite material [5]. The most conductive filaments found were based on metal fillers (e.g., two-dimensional silver powder [6], nickel and tin alloy [7], and silver-coated copper nanowires [8]) with an approximately three-orders-of-magnitude worse conductivity than bulk copper. More common are filaments based on carbon fillers (e.g., carbon black (CB) [9], graphene [10], and carbon nanotubes [11]) that exhibit an additional three orders-of-magnitude worse conductivity than composites with metal fillers.

Polymer nanocomposites are used for sensor fabrication in various applications [12], including biomedical applications [13] and interactive robotics, like electronic skin [14,15]. The piezo-resistive characteristic of conductive FFF materials is often exploited for strain sensing. Maurizi et al. [1] printed a functional small-strain-rate sensor in a dual extrusion process. Kim et al. [16] and Christ et al. [17] researched highly flexible high-strain-rate sensors that can be used in wearable electronics. A fully printed functional accelerometer was presented by Liu et al. [18]. Force-sensing potential was also demonstrated with a multi-axial force sensor, fabricated by Kim et al. [19]. Kwok et al. [9] researched a conductive composite material based on carbon black (CB) and performed electrical, thermal and UV stress tests, before fabricating a functional temperature sensor. Clower et al. [20] printed a Sierpinski tetrahedron-based antenna, where the advantages of additive manufacturing (AM) were utilized for the fabrication of a geometrically complex structure capable of wide-band multi-frequency wireless-signal transmission. In comparison to 3D-printed structures with embedded metal wires [21] and electronic chips [22,23], single-process 3D-printed sensors have the potential to fabricate multi-material functional structures with a structure/property relationship without the need for additional technology or intervention during the fabrication process.

Process parameters have been shown to have an influence on the conductivity of a printed structure. For the Electrifi material [24] used in this work, the manufacturer specifies the right process parameters to attain a higher conductivity of the printed structure, or in other words, to minimize the degradation of the conductive network in the material during the extrusion process. Watschke et al. [3] researched the effects of raster angle, print speed, flow rate, and extrusion temperature on the conductivity and heat-radiation capacity for four different filaments, including the Electrifi. Hampel et al. [25] identified the optimal process parameters (layer height, printing speed, nozzle temperature, and cooling) and derived a conductive model for the electrical circuit’s fabrication. Zhang et al. [26] characterized a CB/ABS composite by measuring the resistivity and its anisotropy for filament, fiber, and cubic samples.

The main focus of this study are the optimal process parameters for highly conductive printed elements (conductors) for the transmission of sensor signals. Although the study is based on previous research [3,25,26], the process parameters are studied for a complete printability value range with high discretization. Five samples were printed for every parameter variation, so the repeatability of the printing process was ensured and the effect of individual printing parameters confidently deduced. The experimental results for six parameters with a combined 41 value variations enabled an insight into the underlying mechanisms, essential for the optimization of the FFF process for a highly conductive structure’s fabrication. Additional experiments with embedded conducting traces were performed to study the effect of the printing of an insulating material on the resistivity. As a whole, this work provides the practical information about the effect the printing process has on the conductivity of the printed structure. It provides the explanation of underlying mechanisms and how the degrading effects can be minimized to attain improved repeatability of the electrical properties and higher electrical conductivity of the printed conductors.

The paper is organized as follows: the researched process parameters, conductive sample fabrication and resistivity measurements are presented in Section 2, followed by the presentation and discussion of the results (Section 3) and conclusions of the study (Section 5).

## 2. Materials and Methods

In this section the presentation of the researched process parameters and the embedding of the conductive tracks is followed by the preparation of the sample and the fabrication of the electrical contacts. The measurement details are presented at the end of this section.

### 2.1. Process Parameters

**Figure 1:**
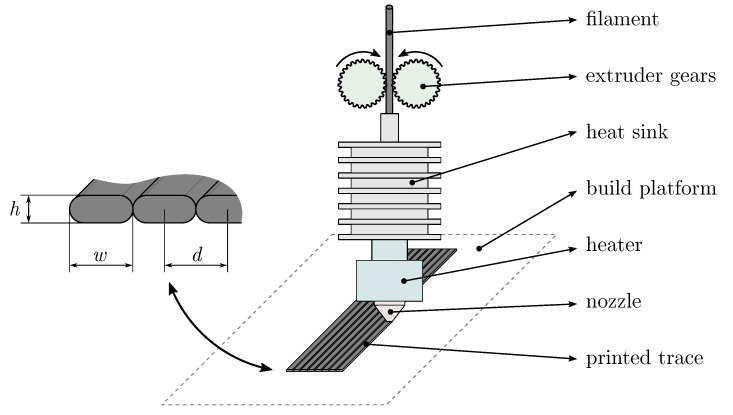
Filament extrusion and printed traces.

The conductive material was oriented parallel to the printing traces, as prior research [3,25,26] showed that the printed conductive samples have the lowest resistivity in this direction. The focus of this study is the influence on the resistivity of the following process parameters: layer height *h*, trace spacing *d*, printing speed *v*, trace width *w* via the extrusion rate, nozzle temperature $T_{\mathrm{nozzle}}$, and bed temperature $T_{\mathrm{bed}}$ (see Figure 1).

The ranges of the six process parameters, determined by the initial printability testing, are given in Table 1.

A total of 41 parameter variations were determined. Each parameter was investigated with the other parameters held constant. The aim of this study was to increase the value range and discretization of the researched parameters. With the determined discretization of five to nine values per parameter, the interactions between the parameters were not studied.

The boundary parameter values were determined with preliminary printability trials. If the extrusion and adhesion of the trace to the printing surface were successful and the trace dimensions were accurate and uniform over the trace length, the parameter variation was included in the study. The Electrifi material exhibits a lower printability than typical FFF filaments like PLA and ABS, because of the high filler concentration, which contributes to a lower flowability in the melted state. The layer height and printing speed were investigated in the typical range of the FFF printing process. For the trace spacing variation, the minimum value (0.25 mm) was determined with the observation of nozzle fouling, when excess material started accumulating on the nozzle. The largest trace spacing was determined so the moving nozzle did not come into contact with the already-deposited material. An extrusion rate lower that 90% resulted in the unsuccessful adhesion of the extruding material on the print surface, and higher than 130% resulted in non-uniform trace dimensions and a low-quality surface of the printed trace. The lowest nozzle temperature at which extrusion was possible was 100 ${}^{\circ }C$. At lower temperatures the extruder gears striped the filament surface and the feeding mechanism failed. Nozzle temperatures higher than 200 ${}^{\circ }C$ were not investigated because of the observed thermal degradation of the material. The range of the print-bed temperature was determined using the capability of the heat bed on the Prusa 3D printer, which was able to accurately hold the surface temperature in the investigated temperature range.

Table 2 shows the values of the constant parameters for each investigated parameter, e.g., samples printed for the study of the nozzle temperature $T_{\mathrm{nozzle}}$ in the range from 100 ${}^{\circ }C$ to 200 ${}^{\circ }C$ were printed at layer height h=0.2mm, trace spacing d=0.55mm, printing speed v=10mm/s, etc.

Parameters like layer height, nozzle and print-bed temperature, and trace width remained constant for every sample batch. Trace spacing, printing speed and extrusion rate were adjusted for printability reasons, so the range for the researched parameter could be expanded. The trace spacing was increased to 0.65 mm for the layer height and the extrusion rate sample batch so the thicker traces were not in contact. For the bed temperature the batch trace spacing was decreased to 0.40 mm. This was done to investigate whether the negative thermal effect on the printed traces was reduced at certain print-bed temperatures (see Section 3). For the nozzle-temperature sample batch, the printing speed and extrusion rate were reduced to enable extrusion at lower temperatures.

**Figure 2:**
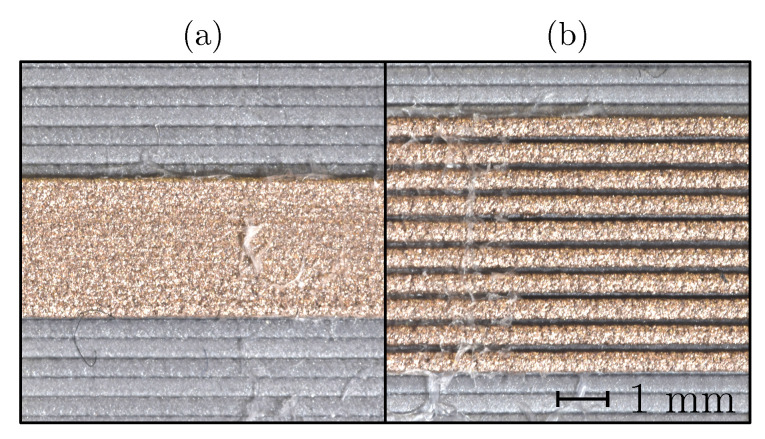
Trace spacing *d* influence: (**a**) d=0.25 mm, (**b**) d=0.55 mm.

The effect of the trace-spacing variation on the shape of the printed samples is evident in Figure 2. At a low trace spacing the printed traces fused and formed a single conducting strand (Figure 2a). At a higher trace spacing the contact between the printed traces was eliminated (Figure 2b). The section area of the printed conductors at varying trace spacing was constant, but the volume requirement increased with the increased trace spacing.

**Figure 3:**
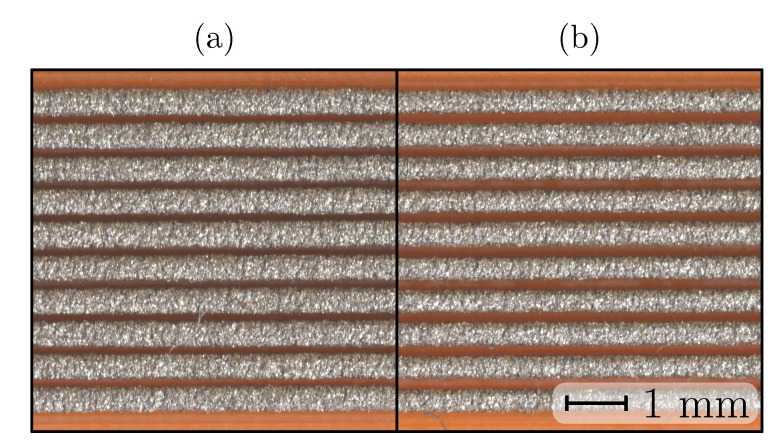
Printing speed *v* influence: (**a**) v=10 mm/s, (**b**) v=40 mm/s.

The effect of printing speed on the shape of the printed samples is shown in Figure 3. Traces printed at high printing speeds exhibited less spread after extrusion (Figure 3b). This was a consequence of the decreased effect of the thermal conduction from the nozzle through the extruding material and increased cooling rate [27].

**Figure 4:**
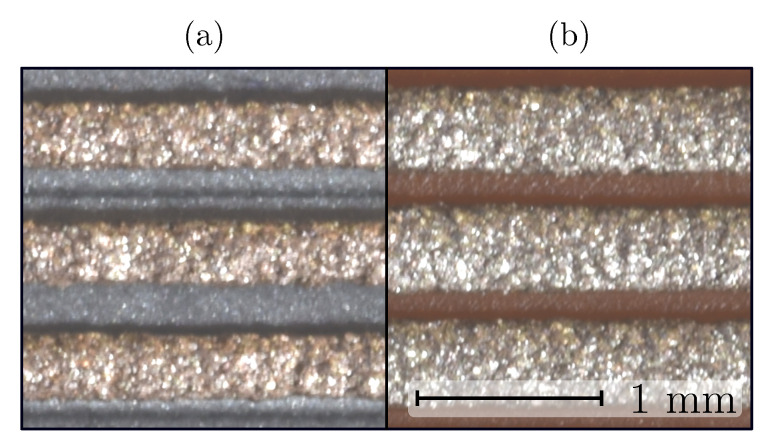
Extrusion rate $E_{r}$ influence: (**a**) $E_{r}=90\;\%$, (**b**) $E_{r}=130\;\%$.

As was expected, the extrusion rate affected the width of the printed traces (see Figure 4). The change in the trace width affected the air gap between the traces. For that reason the trace spacing for the samples printed with varying extrusion rates was increased to 0.65 mm (see Table 2) to ensure no contact between the neighboring traces and no contact between the already-printed traces and the moving nozzle occurred during printing. At extrusion rates of 120% and 130% a reduction of the surface quality was observed. An increased extrusion rate increased the pressure and shear rate in the nozzle narrowing [27]. In conjunction with the low printability of the Electrifi material, this produced rough trace surfaces.

**Figure 5:**
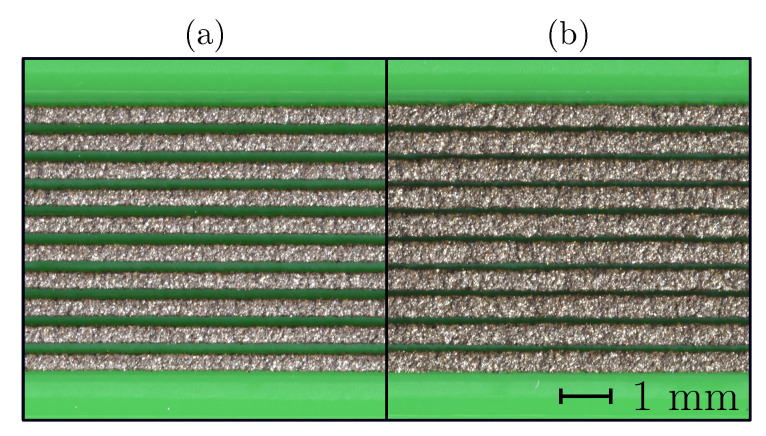
Nozzle temperature $T_{\mathrm{nozzle}}$ influence: (**a**) $T_{\mathrm{nozzle}}=100{\;}^{\circ }C$, (**b**) $T_{\mathrm{nozzle}}=200{\;}^{\circ }C$.

The nozzle-temperature variation also affected the shape of the printed traces, as shown in Figure 5. Traces extruded at $T_{n}=100{\;}^{\circ }C$ exhibited no spread after the extrusion (Figure 5a). Traces extruded at $T_{n}=200{\;}^{\circ }C$ exhibited substantially lower viscosity and deformed after the extrusion with an about 25% increase in trace width compared to the traces printed at $T_{n}=100{\;}^{\circ }C$ (Figure 5b).

**Figure 6:**
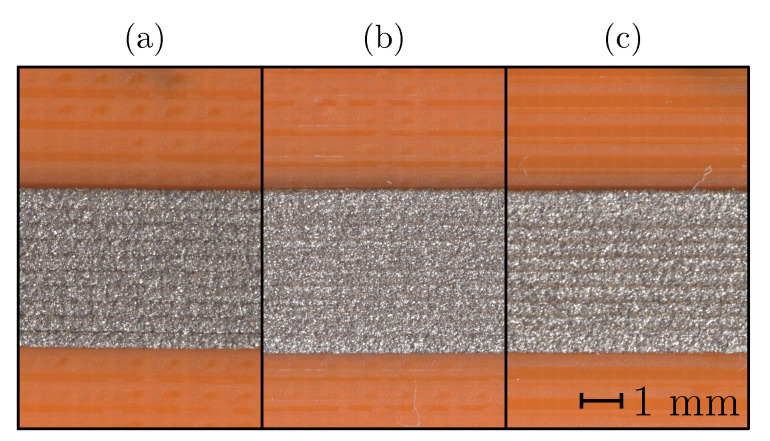
Bed temperature $T_{\mathrm{bed}}$ influence: (**a**) $T_{\mathrm{bed}}=30{\;}^{\circ }C$, (**b**) $T_{\mathrm{bed}}=60{\;}^{\circ }C$, (**c**) $T_{\mathrm{bed}}=90{\;}^{\circ }C$.

The variation of the print-bed temperature did not produce any visual differences between the printed samples (see Figure 6) (color differences are a consequence of the lighting conditions). The decreased trace spacing (see Table 2) for the print-bed temperature investigation is commented on in Section 3.

Although Figure 2, Figure 3, Figure 4, Figure 5 and Figure 6 exhibit some differences in surface morphology between the different colors of PLA board (printing surface), the differences were negligible and were determined not to influence the electrical properties of the conductive traces.

### 2.2. Embedded Conductive Tracks

In addition to the process parameters (Table 1), in real-world applications, conductive elements are usually embedded into a dielectric surrounding material, functioning as a structural support. Furthermore, materials in contact with conductive elements could function as heat-transmitting elements for cooling or heating the structure.

**Figure 7:**
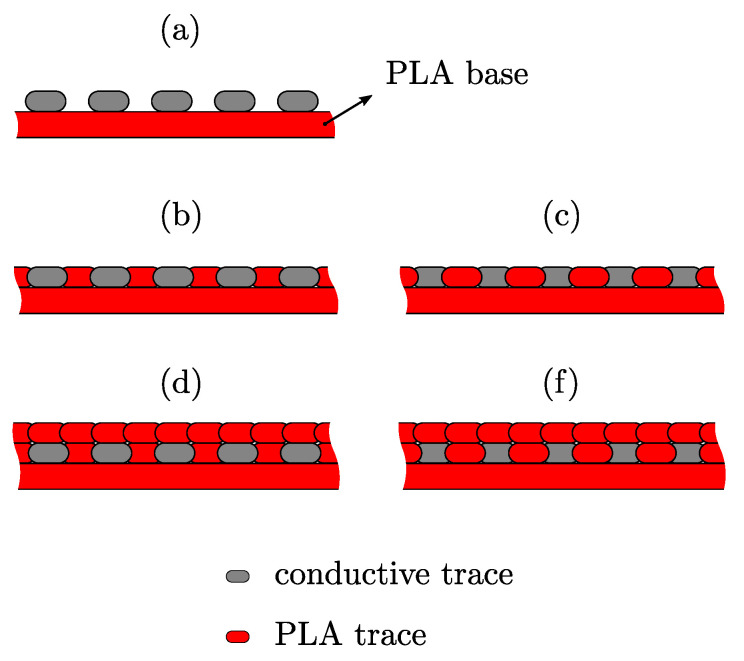
Different embedding of conductive and insulating traces: (**a**) without PLA insulation, (**b**) insulating PLA traces between conductive traces (conductive traces printed first), (**c**) insulating PLA traces between conductive traces (insulating traces printed first), (**d**) variation of (**b**) with insulating overprint, (**e**) variation of (**c**) with insulating overprint.

In this study, five different variations of the conductive-material embedding were researched: Figure 7a without PLA insulation, Figure 7b first conductive, then insulating traces, Figure 7c first insulating, then conductive traces, Figure 7d variation of Figure 7b, but with an insulating overprint, Figure 7e variation of Figure 7c, but with an insulating overprint.

**Figure 8:**
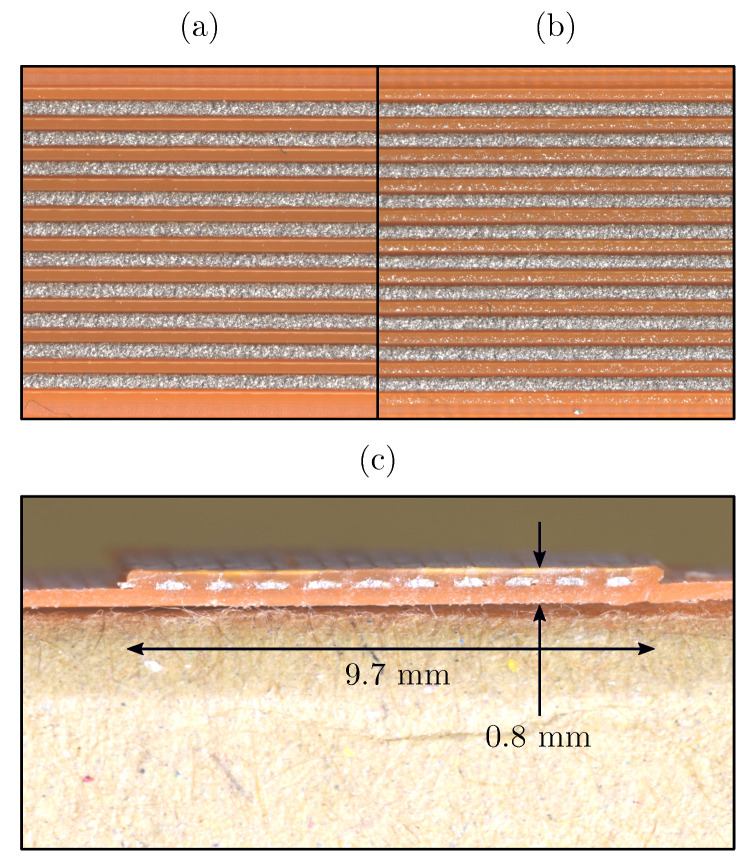
Embedded conductive traces: (**a**) corresponding to Figure 7b, (**b**) corresponding to Figure 7c; (**c**) section of a sample with PLA overprint.

Although the porosity of the multi-material FFF structures was not the focus of this study, the process parameters were adjusted to reduce the voids between the traces (see Figure 8) so that the thermal contact between the individual traces and its effect on the conductive properties could be investigated.

Conductive traces were printed at: d=0.9 mm, v=20 mm/s, $E_{r}=110$%, $T_{\mathrm{nozzle}}=140{\;}^{\circ }C$. The build platform (print bed) was at room temperature during the printing. The layer height also remained constant at 0.2 mm. Insulating PLA traces were printed at: d=0.9 mm, v=35 mm/s, $E_{r}=80$%, and $T_{\mathrm{nozzle}}=200{\;}^{\circ }C$. The last two sample variations included a PLA overprint, which was printed at: d=0.45 mm, v=35 mm/s, $E_{r}=150$%, and $T_{\mathrm{nozzle}}=215{\;}^{\circ }C$.

### 2.3. Sample Printing

Samples were printed with a Prusa i3 MK3S 3D printer. A brass nozzle with a hole diameter of 0.4 mm and a standard spring-steel sheet with a polyethyleneimine (PEI) coating as a build platform were used.

Conductive samples were printed with an Electrifi conductive filament (filament resistivity ρ=0.006 Ω cm), a composite of biodegradable polyester (PCL) and copper nanowires [24].

**Figure 9:**
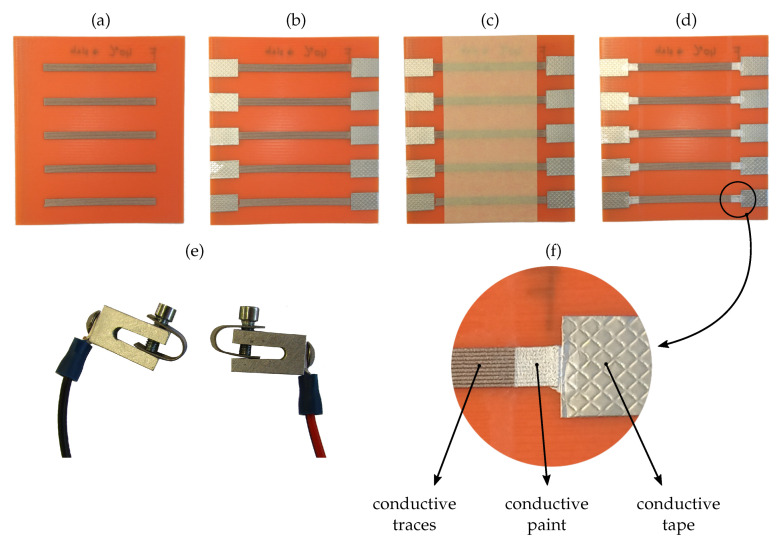
Preparation of electrical contacts: (**a**) printed sample board, (**b**) application of conductive tin-plated copper foil, (**c**) application of protective 50 mm tape, (**d**) application of silver conductive paint, (**e**) contacting claps, (**f**) magnified electrical contact.

For better repeatability of the sample preparation, the samples were printed on a rectangular base with a 100% rectilinear infill pattern, ensuring structural stiffness and protection (see Figure 9a). The dimensions of the sample board were 90×100×0.4 mm. A low thickness was required to enable the investigation of the print-bed temperature on the conductive traces (PLA board acted as an insulator). The length of the printed conductive samples was 60 mm and the effective conducting length was 50 mm, since 5 mm on each end was used for the contacting (see Figure 9).

The STL file for the PLA board onto which the samples were printed was generated with SolidWorks and the G-code for PLA board was generated with PrusaSlicer. Standard PLA filaments (Plastika Trček d.o.o.) in grey, orange, and green were used as structural and dielectric materials. The reported DC resistivity of the PLA printed material was $10^{13}$ Ω cm [28] i.e., high enough to be used as an insulator. No effect of the color filler on the conductivity of the printed traces was found in the preliminary measurements.

The G-code for the conductive samples was generated with a custom Python script based on Equation (2). The G-code for the PLA board and the G-code for the conductive samples were combined with the Python script. The developed Python code is accessible at GitHub [29].

The extrusion control in the G-code generation program was based on:

(1) $$L_{f}\;\pi \;\frac{d_{f}^{2}}{4}=L_{t}\;\left(\pi \;\frac{h^{2}}{4}+\left(w-h\right)\;h\right),$$

where the left-hand side represents the volume of the used filament and the right-hand side the volume of the printed trace (thermal deformations and material loss were neglected) [27]. In Equation (1) $L_{f}$ is the filament length, $d_{f}$ is the filament diameter, and $L_{t}$ is the trace length. With specified trace dimensions (width *w*, height *h* and length $L_{t}$) and a known filament diameter $d_{f}$, the length of the required filament $L_{f}$ is:

(2) $$L_{f}=E_{r}\;L_{t}\;\frac{4\;\left(w-h\right)\;h+2\pi \;h^{2}}{\pi \;d_{f}^{2}},$$

where $E_{r}$ is the extrusion rate.

For each parameter value, five identical samples were printed on a shared PLA sample-board, as shown in Figure 9a. The 41 sample variations and five samples printed for each variation resulted in 205 samples in total, excluding the preliminary printability tests (for defining the parameter ranges, see Table 1 and Table 2), samples with embedded conducting traces (Figure 7) and samples for the investigation of the post-printing heating.

The sample boards were printed with two different materials. Because of the single extruder on the used FFF 3D printer, a manual filament change was necessary. To ensure that no material contamination occurred, the following procedure was utilized. After the PLA board was printed with the PLA filament, the extruder head was lifted to the top position and the PLA filament was unloaded. The material remaining on the exterior nozzle surface was cleaned off. Next, the Electrifi filament was loaded and extruded through the nozzle until the inside of the extruder was free of PLA melt and clean Electrifi melt was extruding. The nozzle temperature was adjusted and additional material was extruded to ensure no over-heated material remained in the extruder. If the procedure was successful, the nozzle was lowered back to the printing surface and conductive sample printing commenced. In the case of embedded samples, two manual filament-change procedures were necessary to print one sample board.

### 2.4. Electrical Contacts

The electrical contact, fabricated between an electrically conductive printed part and the measurement circuit, is shown in Figure 9. First, the conductive traces were printed on the sample-board, see Figure 9a, then the 3M embossed tin-plated copper foil (1345) tape was applied [30] (Figure 9b), followed by the application of a 50-mm-wide protective tape (Figure 9c), which was used for the controlled application of the Electrolube silver conductive paint SCP03B [31] (Figure 9d). Figure 9e shows the aluminum clamping connectors and Figure 9f the magnified electrical contact. In accordance with the instructions, the silver paint was dried for 24 h at room temperature in dry storage. The conductive paint was approximately 20 times and the copper foil approximately 100 times more conductive than the printed Electrifi material. All the contacts were fabricated with the same process and materials, and consequently contributed an equal amount to the electrical resistance of every sample.

Resistivity measurements were implemented with a two-point contact method [3,32]. The contact resistance of the combined conductive silver paint (CSP), copper foil, contact clamps, and copper conductors was consistently measured at 0.1 Ω, while the average resistance of all the printed samples was 4.8 Ω. The resistance of the contacting materials was measured on special samples, fabricated with CSP, copper foil, clamps and copper wires of the same length as were used in the experiments.

### 2.5. Resistivity Measurement

**Figure 10:**
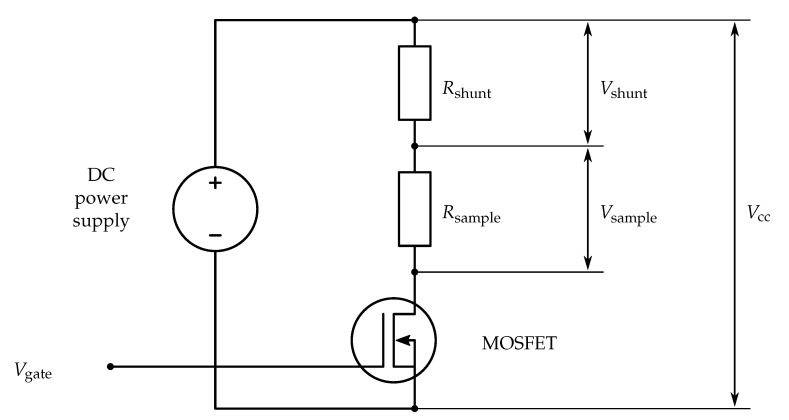
Measurement circuit.

The measurement circuit is shown in Figure 10. The voltage drops on the shunt resistor (5 Ω) and the connected sample were measured with a National Instruments (NI) 9215 data acquisition (DAQ) card. As a power source, a Voltcraft DPPS-60-10 DC power supply was used.

An important aspect of the resistance measurement of FFF-printed conductive structures is that the resistance can be very dependent on the temperature of the material [9]. Even at a constant environmental temperature, the resistive heating can change the spot temperature inside the material (and therefore the conductivity). To prevent resistive heating, the excitation voltage was relatively low (1.5 V), (the voltage drop across the sample was approximately 0.7 V). On average, a voltage of 0.7 V generated a current of 0.15 A. A current of approximately 0.3 A was needed for a local temperature increase of more than 1 ${}^{\circ }C$. The temperature of each sample was measured with a type-K thermocouple and a NI 9211 DAQ card. The typical temperature increase of 0.5 ${}^{\circ }C$ during a 30 s measurement was determined to have a negligible effect on the resistivity. A MOSFET was used to control the measurement time (see Figure 10), operated by an NI 9260 analog output card.

The resistivity of each sample was determined from the measured resistance and the known trace dimensions. The trace dimensions were not measured manually but were determined from the G-code, generated for each sample. The volume of extruded material *V* for each sample was calculated using the known filament extrusion length $L_{f}$ and diameter $d_{f}$. The section area was calculated by dividing the volume by the sample length $L_{s}$:

(3) $$A_{s}=\frac{V}{L_{s}}=\frac{L_{f}\;\pi \;\frac{d_{f}^{2}}{4}}{L_{s}}.$$

This method used for the section-area calculation was determined to be sufficient, since the default accuracy of the Prusa MK3S FFF 3D printer was 10, 10, 2.5, 3.6 μm for the X, Y, Z, E axis, respectively, where E is the extruder feed drive [33]. In addition, the accuracy and repeatability of the printing process were verified on test samples before the study and no loss of material (nozzle fouling) was ensured during the printing process.

With the determined section area $A_{s}$ and the known conducting length of the sample $L_{c}=50$ mm, the resistivity was calculated:

(4) $$\rho_{s}=R_{s}\;\frac{A_{s}}{L_{c}}.$$

## 3. Results

**Figure 11:**
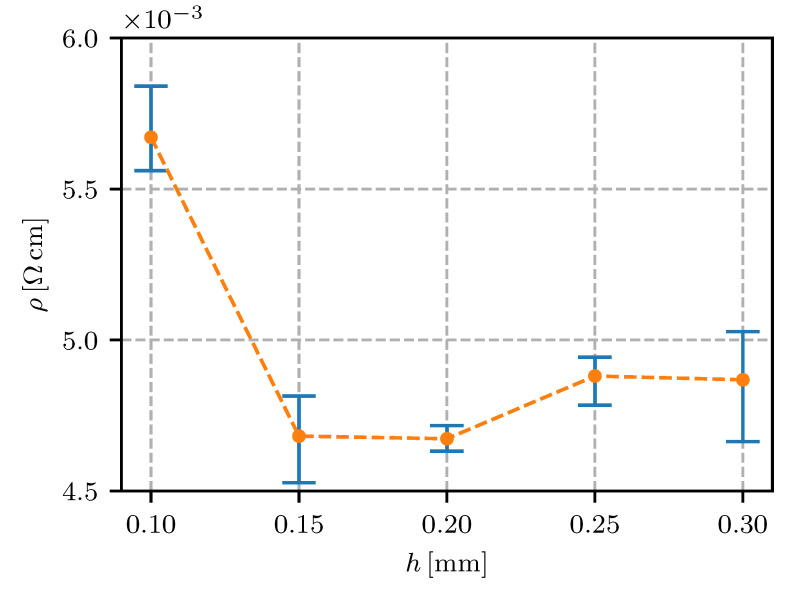
Resistivity for different layer heights *h*.

**Figure 12:**
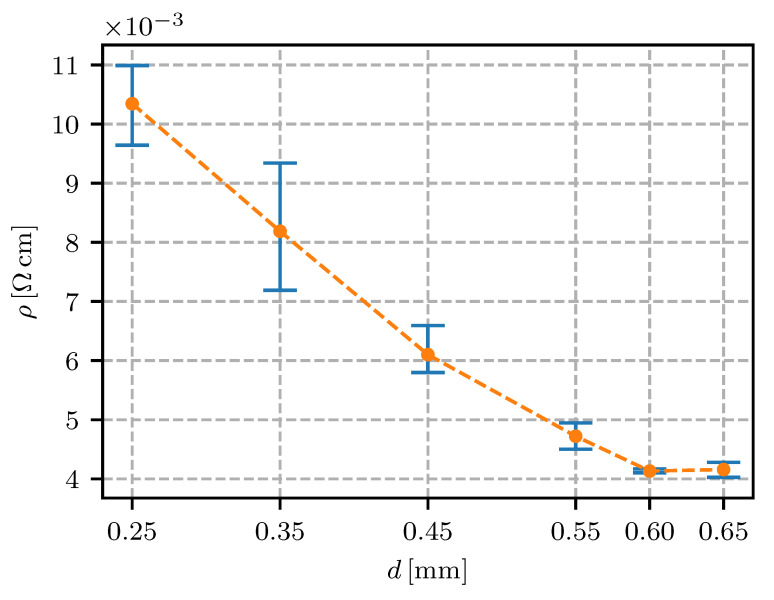
Resistivity for different trace spacings *d*.

**Figure 13:**
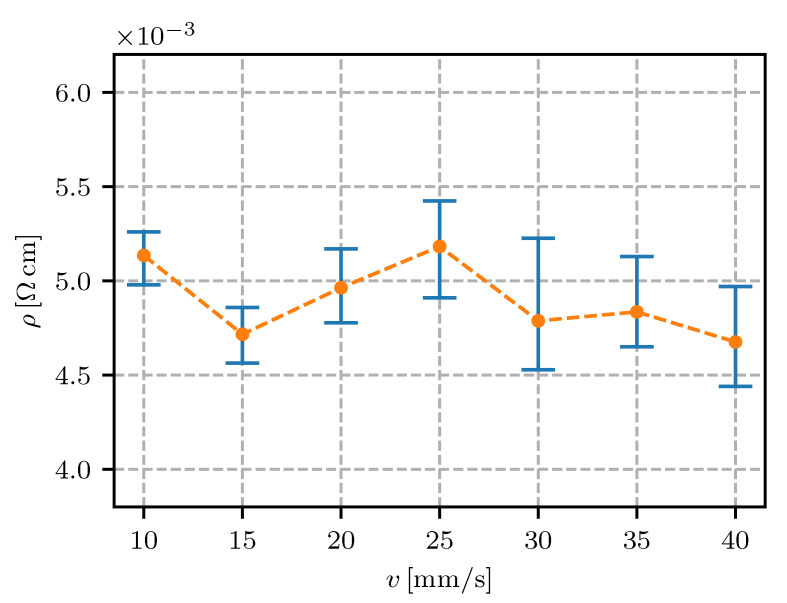
Resistivity for different printing speeds *v*.

**Figure 14:**
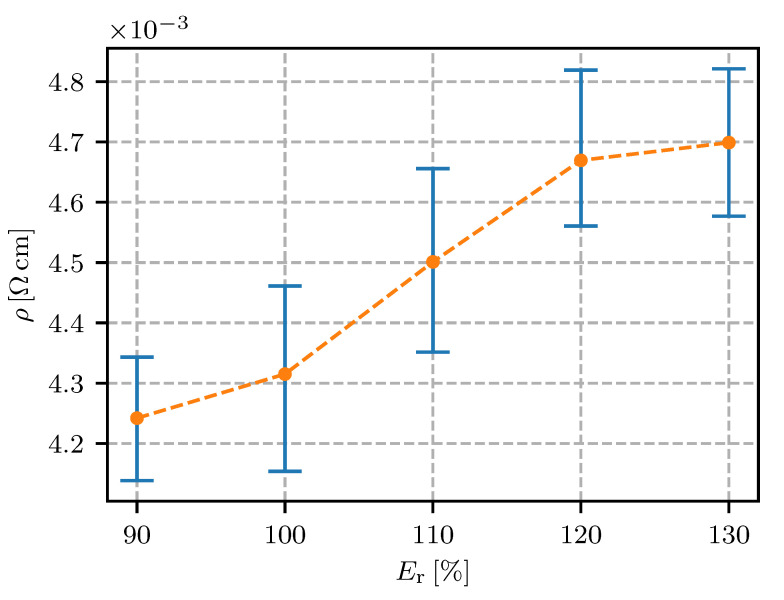
Resistivity for different extrusion rates $E_{r}$.

**Figure 15:**
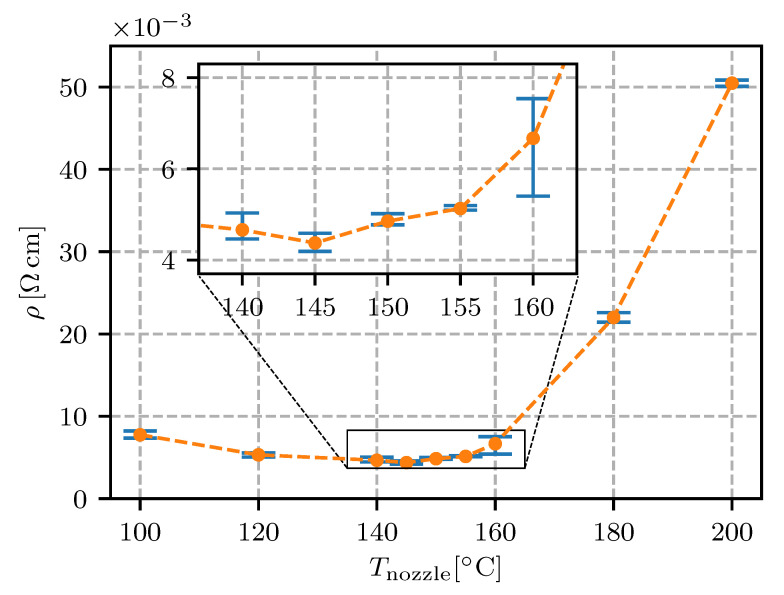
Resistivity for different nozzle temperatures $T_{\mathrm{nozzle}}$ with an enlarged optimal range.

**Figure 16:**
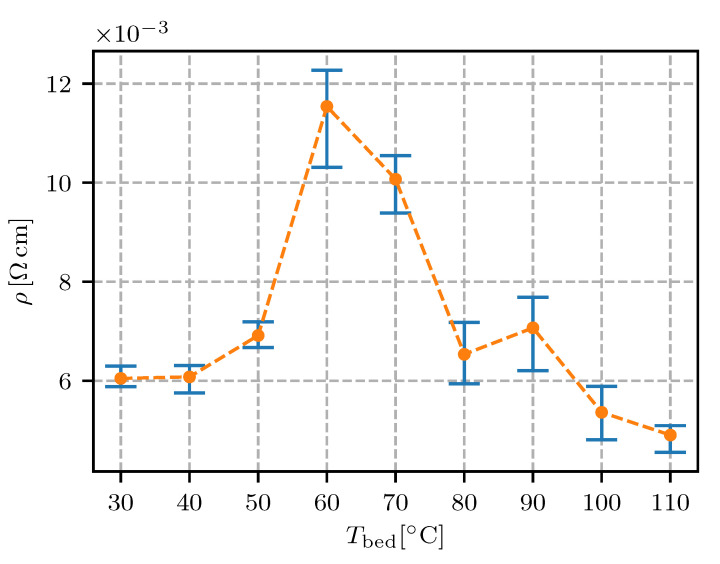
Resistivity for different print-bed temperatures $T_{\mathrm{bed}}$.

**Figure 17:**
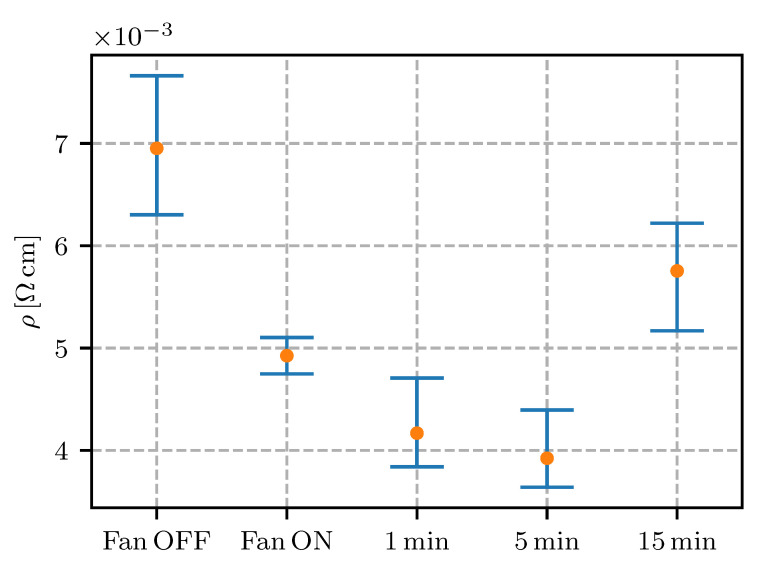
The effect of active cooling during the printing and melting phase after the printing process.

**Figure 18:**
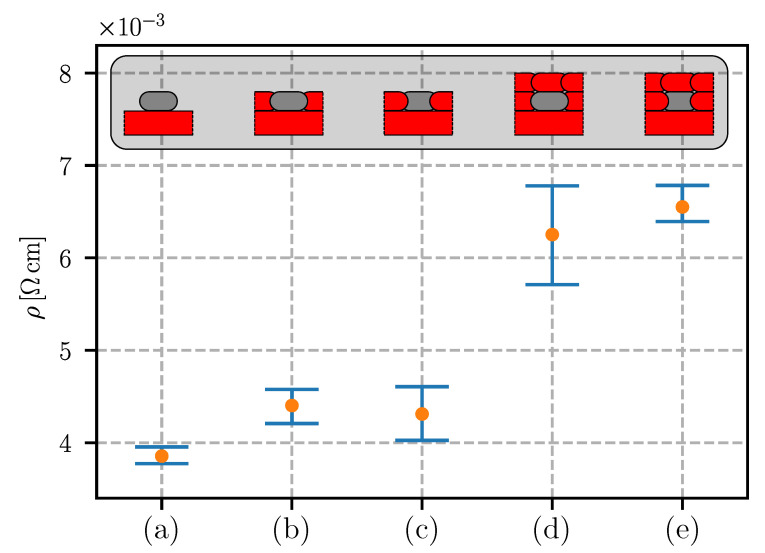
Effect of PLA insulating traces (letters corresponding to Figure 7); sample sections are added for clarity.

The results are presented as the resistivity (i.e., the inverse of the conductivity) plotted against the values of each defined parameter (see Table 1). In the following figures (Figure 11, Figure 12, Figure 13, Figure 14, Figure 15, Figure 16, Figure 17 and Figure 18) the point represents the average of five samples printed with identical parameters, while the error bars represent the range from the lowest to the highest measured value. The results in statistical form are also presented in Table 3 with included mean resistivity and standard deviation of resistivity measurement for every parameter variation.

The results from the layer-height variation (Figure 11) showed 0.15 to 0.2 mm to be the optimum layer height. A greater layer height than the default 0.2 mm increased the resistivity by less than 5%, and a lesser layer height increased the resistivity by 20%. The increased resistivity for a lower layer height could be due to the decreased printing quality and increased surface-to-volume ratio of the sample.

The increased deformation of the extruding material in conjunction with the low storage modulus and high viscosity of the Electrifi material [24] contributed to a reduced surface quality of the printed traces. An uneven and cracked surface reduced the effective conducting section area of the sample and increased the resistivity.

Electrifi and other metal/polymer nanocomposites suffer from oxidation of the filler particles, which is accelerated at high temperatures of the FFF process [3,7,8]. Oxidation of the exposed material increases the resistance of the filler particles and can break the conducting connections, so increasing the resistivity of the composite system. Reducing the layer height for a constant trace width increases the surface/volume ratio of the trace. That means more of the material is exposed to the atmosphere and the external nozzle surface, and the negative effect of thermo-oxidation is increased.

The trace spacing is one of the parameters that showed significant potential for resistivity reduction. Increasing the gap between the traces substantially reduced the resistivity (see Figure 12), reaching the lowest resistivity when the air gap between the traces ensured no contact between the already-deposited traces and the moving nozzle occurred during the printing (see Figure 2).

In conventional FFF printed structures, the distance between the individual traces is set so the porosity is minimal, since this negatively effects the mechanical properties of the printed structure. Tightly deposited traces enable increased diffusion of the polymer chains in the contact region, which increases the weld strength and consequently the strength of the whole structure.

In terms of the electrical properties of the Electifi material, contact between the individual traces reduced the electrical performance. The degrading effect of the thermal and mechanical contacts between the deposited and extruding material (and the nozzle) can be attributed to the increased cooling time of the material and the mechanical deformations of the deposited traces. An increased cooling time translated into a longer time in the temperature range of accelerated oxidation. In addition to the increased oxidation, the deposited traces experienced secondary deformation as the extruding material heated and deformed the solidified traces. This deformation could break the conducting connections in the material and increase the resistance of the printed structure. In addition, if the parameter was set too low, the tightly packed extruding material could cause material buildup on the nozzle surface, reducing the surface quality and the conducting section area and ultimately increasing the resistance of the printed conductor.

When the trace spacing was increased over 0.55 mm the contact between the nozzle or extruding material and the deposited material was prevented, as can be observed in Figure 2b). This produced the lowest resistivity for this sample group, at $4\times 10^{-3}$ Ω cm (see Table 3).

The results showed that in terms of material consumption, the most effective conductor was printed with a trace spacing wide enough to eliminate any contact with the deposited material. In tightly packed, multi-material structures with embedded conductors, a wide trace spacing and an increased volume consumption might not be the best solution. With the experimentally determined effect of the trace spacing, the printing process can be optimized for the resistivity and conductor volume requirements.

Printing-speed variations in the investigated range only contributed 5% to the resistivity deviation from the nominal filament resistivity. The results in Figure 13 and Table 3 show a slight increase in the measurement scatter with a printing speed faster than 20 mm/s.

The Electrifi material, because of its high filler content, exhibited a higher viscosity than the typical FFF polymers like PLA and ABS. The high viscosity and low storage modulus were more evident at high printing speeds, when the material extrusion and adhesion to the print surface became unstable. Lower stability of the process increased the scattering of the material properties, including the resistivity, which can be observed in Figure 13 and in Table 3.

In Figure 3 the effect of printing speed on the air gap between the traces can be observed. The effect of trace spacing (air gap) on the resistivity was determined to be significant (see Figure 12), while the change in air gap with printing-speed variation was not evident. The effect of the trace spacing parameters was in the thermal contact between the already-deposited material and the extruding material and the nozzle. At various printing speeds the trace width and consequently the air gap between the traces changed, but no thermal contact occurred. For that reason, a change in the air gap (trace spacing) did not have an effect on the resistivity of the samples printed at various printing speeds.

The results of the extrusion-rate variation showed that a lower extrusion rate lowered the resistivity (see Figure 14 and Table 3).

The cause for this might be the viscous flow conditions in the nozzle. Increasing the extrusion rate required a higher filament-feeding force, which increased the pressure and shear rate in the nozzle narrowing. Higher shear rates are known to break apart the clusters of the filler particles in the nanowire and nanotube polymer composites [34]. Breaking the conducting network increases the resistivity on the macro scale. In addition, a fast extrusion rate deforms the extruding trace with material overflow, which can break apart the conducting nanoparticle clusters and increase the resistivity.

The effect of nozzle temperature on the resistivity was expected. The manufacturer specified that the optimal range for the extrusion of an Electrifi filament was between 130 and 160 ${}^{\circ }C$ [24]. This was confirmed by our experiment and the optimal temperature of 145 ${}^{\circ }C$ for our setup was determined (see Figure 15 and Table 3).

The results showed, that the thermo-oxidation of copper nanowires in the Electrifi material was most pronounced at nozzle temperatures over 160 ${}^{\circ }C$. At nozzle temperatures of 180 and 200 ${}^{\circ }C$ the resistivity was 5- and 12-times higher, respectively. In contrast to the effect of oxidation, a lower nozzle temperature also increased the resistivity (∼80% increase at $T_{\mathrm{nozzle}}=100{\;}^{\circ }C$). The lower temperature of the polymer composite melt in the extruder caused a higher viscosity. The higher viscosity of the flowing polymer at a constant volumetric flow rate increased the shear stress in the nozzle. The shear stress damaged the microstructure (breaking the conductive nanowire clusters and the individual nanowires) of the composite system. The optimal nozzle temperature was, therefore, a compromise between the desired viscosity of the polymer melt and a minimized time in the high-temperature range of increased oxidation.

The results of print-bed temperature variation showed a range from 50 to 90 ${}^{\circ }C$ to be avoided (see Figure 16). At 60 ${}^{\circ }C$, which is the melting point of the Electrifi composite [24], the resistivity increased by 100%. At temperatures over 80 ${}^{\circ }C$, the resistivity dropped back to the initial values, while the measurement scatter remained high (better observable in Table 3).

The results present an insight into the interplay of multiple mechanisms of degradation and formation for the conductive network in the polymer matrix. The effect of these mechanisms was dependent on the temperature of the material.

At print-bed temperatures around 60 ${}^{\circ }C$ the degrading effect on the conductivity was evident. With an increased cooling time for the extruding material, thermo-oxidation was more pronounced and the samples exhibit an increased resistivity. With an increased print-bed temperature, the negative effect of the thermo-oxidation was increased. Contrary to this notion, the negative effect of oxidation was reversed at $T_{\mathrm{bed}}>70{\;}^{\circ }C$. Therefore, a previously unnoticed mechanism of conductive-network formation must have been at play in this temperature range.

A study of the conductive network’s formation in polymer nanocomposites has shown that the formation of the conductive network is possible when the matrix material is in a liquid phase and nanoparticles can diffuse between the polymer chains. Nanoparticles and interparticle attractive forces can form new conducting pathways, decreasing the resistivity on the macro-scale [34].

In practical terms the results of print-bed temperature show that when the extruded material was kept completely melted for the time of printing, copper nanowires (and clusters of nanowires) in the polymer melt agglomerated and formed secondary clusters, which decreased the resistivity of the printed structure.

Samples for the print-bed temperature experiment were printed without an air gap (see Table 2 and Figure 6). The results of trace spacing (air gap) showed that the contact between the deposited traces increased the resistivity (see Figure 12), which was confirmed with the samples for print-bed temperature at 30 and 40 ${}^{\circ }C$. The results showed that the negative effect of a low trace spacing could be reversed at print-bed temperatures of 100 and 110 ${}^{\circ }C$.

To confirm that the resistivity reduction is possible after the extrusion and that the conductive network can form new connections, an additional experiment was performed. Printed samples were kept on the print surface after the printing and the temperature of the heat bed was increased from 40 ${}^{\circ }C$ (during printing) to 110 ${}^{\circ }C$ (after printing). During the melting phase, the temperature of the samples was measured to be around 105 ${}^{\circ }C$ (∼5${\;}^{\circ }C$ drop because of convection with the ambient air). The time of exposure was set to 1, 5 and 15 minutes. As a control, two sample boards were printed: one with active cooling during extrusion and one without. The resistivity measurement results are shown in Figure 17 and in Table 3.

It was evident that an optimal time of exposure existed as the mechanisms of conductive network formation through nanoparticle motion and thermo-oxidation affected the conductive properties of the material.

No active cooling during the printing resulted in a higher resistivity, as was expected. Active cooling without the melting phase after printing resulted in samples with the expected nominal resistivity of $5\times 10^{-3}$ Ω cm. If the samples were subjected to the melting phase after the printing the resistivity decreased at 1 and 5 min and increased at 15 min of exposure. One of the samples exposed to 5 min melting phase exhibited the lowest resistivity achieved in this study, i.e., $3.6\times 10^{-3}$ Ω cm.

The results of the sample heating after printing showed that the network formation had a superior effect on the resistivity for 5 min. After 5 min the conductive network formation slowed and oxidation becomes the influencing factor in conductive-network degradation, resulting in a substantially increased resistivity at 15 min. To further determine the material’s optimal time period in the melted state, additional experiments with varying print-bed temperatures and a higher discretization of the heating time period should be performed.

Figure 18 shows the results of embedding the conductive filaments. It was found that the insulating traces, printed in between and over the conducting traces, increased the resistivity by approximately 70%. The resistivity of the embedded traces increased because of the thermal diffusion between the extruding PLA and the deposited Electifi traces. The extruding PLA ($T_{\mathrm{nozzle}}$ = 200–215 ${}^{\circ }C$) heated the Electrifi traces and accelerated the oxidation of the copper filler. In addition, the heated traces deformed under the increased temperatures and loads of the extruding material. The deformation of the matrix polymer broke apart some of the connected clusters of conducting filler, consequently increasing the resistivity of the trace.

## 4. Discussion

The results showed an interplay of the multiple influential mechanisms of degradation and formation of the conductive network in the material. Thermo-oxidation was shown to be the most degrading for the conductive properties of the Electrifi material. Although the oxidative degradation is most prominent at nozzle temperatures over 160 ${}^{\circ }C$, its effect is also noticeable at lower nozzle temperatures, when the print-bed temperature is increased and the traces experience secondary thermal contact with the extruding material or the nozzle. Therefore, the oxidative degradation of the electrical performance can be minimized with optimal printing temperatures, but also with active cooling of the extruding material and reduced thermal contact between the deposited and extruding material (large trace spacing). The trace-spacing adjustments can lower the resistivity by an additional 20%.

Another degradation mechanism was most noticeable with the extrusion rate variation. The extent of the damage of the established conducting network in the filament is also dependent on the pressure and shear rate in the nozzle narrowing [34]. Shear degradation of the conductive network can be minimized with low printing speeds and extrusion rates, which can conversely increase the time of the high temperature exposure and consequent oxidation. The material flow rate (as a function of trace dimensions, printing speed, and extrusion rate) must therefore be experimentally determined as a compromise between the oxidative and shear degradation of the conductive network. The results showed that when printing at the optimum nozzle temperature of 145 ${}^{\circ }C$ and at a low extrusion rate, the printing speed can contribute an additional 10% to the resistivity reduction. The shear stress and temperature of the material in the extruder is dependent on the internal shape, surface and material of the extruder and the nozzle [27,35]. Therefore, the optimum parameters can deviate for different FFF printers and nozzle configurations.

Another mechanism was discovered with the variation of the print-bed temperature. The diffusion of nanoparticles and nanoparticle clusters in the polymer melt can be utilized for a decrease of the resistivity after the printing process, without technological modification or manual intervention. An additional experiment showed that this mechanism can have a contribution of ∼20% resistivity reduction to already optimally printed samples, reaching a resistivity of $3.6\times 10^{-3}$ Ω cm, which is 40% lower that the filament resistivity specified by the manufacturer [24]. In the case of thin conductors in close proximity to the print surface, the heat bed can be utilized for the melt phase after the printing process. Additionally, the printed conductors have to be supported to prevent the deformation of the melted material. The necessary support and the problem of heating of the embedded conductors are the main limitations in a resistivity decrease for the melting phase after the printing.

Although layer height, trace spacing, extrusion rate, printing speed, nozzle temperature, and print- bed temperature have a coupled effect on the discovered conductivity degradation/formation mechanisms, an optimal printing strategy can be deduced. Primarily, the printability of the material in a specific parameter range has to be ensured. As multiple researchers have already found that oxidation is the main factor in a resistivity increase in metal/polymer nanocomposites (excluding noble metals) [3,7,8], it has to be minimized to retain the conductive properties of the filament material. Since high temperatures accelerate the oxidation process [36], low nozzle temperatures and increased cooling of the extruding material can reduce the filler oxidation. Further, a low printing speed and extrusion rate reduce the shear rate in the nozzle narrowing and reduce the damage of the established conductive network during the extrusion. Furthermore, resistivity can be reduced with the melting of the conductive material after the printing process (if this is technologically possible) enabling the formation of new conducting pathways. The optimum time in the melted state has to be determined with preliminary tests, as the effect of network formation and oxidation are dependent on the conductive material (filler and polymer matrix properties), the ambient conditions and the shape of the printed structure.

The process parameters have a coupled effect on the flow (shear), thermal, and phase state of the composite system. To advance our knowledge of influential mechanisms, the effects of the process parameters have to be decoupled. Shear degradation of the filler nanoparticles with a high aspect ratio (wires, tubes, rods) can be studied in conjunction with models for polymer melt flow in the extruder [27,35] to decouple the effect of nozzle temperature (influencing the viscosity) and material flow rate (dependent on trace dimensions, extrusion rate, and printing speed) on the shear stress in the extruding material.

The oxidation of copper nanowires and other metal nanoparticles was already thoroughly studied [36,37] and practical solutions exist, mostly in the form of coatings with ligand molecules [38] and inert metals [8].

The promise of conductive-network formation was already discussed in research literature [34]. The mechanism is driven by the thermal and shear state of the polymer melt. Finding the optimal thermal state for increased nanoparticle diffusion and secondary agglomeration after the extrusion process can be determined for a specific material with a thermal model of the conducting structure in conjunction with resistivity measurements.

## 5. Conclusions

This study presents the influence of six process parameters (layer height, trace spacing, printing speed, extrusion rate, nozzle temperature, and print-bed temperature) on the resistivity of printed conductive samples in a unidirectional layout.

For electrical conductors in fully-embedded sensors (sensors as well as the connecting wires and support structures are 3D fabricated) it is essential to have the wire’s resistivity under control. In this research it was found that the height of the printing layer should be in the range from 0.15 mm to 0.30 mm; a 0.1 mm or lesser height is expected to significantly increase the resistivity. The resistivity for the conductive trace spacing was found to decrease if the spacing was increased, reaching a minimum at 0.6 mm. The printing speed in the range from 10 mm/s to 40 mm/s was not found to have a significant influence on the resistivity. With regard to the extrusion rate, the lowest resistivity was found to be 90% (lower values would impact on the quality of the printing). The temperature of the nozzle was found to have a minimum at 145 ${}^{\circ }C$, with a temperature window of +/−10 ${}^{\circ }C$ where the resistivity was still relatively low. The effect of heat-bed temperature was found to significantly increase the resistivity at 50 ${}^{\circ }C$ to 80 ${}^{\circ }C$, while the heat-bed temperature ranged from 30–40 ${}^{\circ }C$ or 100–110 ${}^{\circ }C$ were found to decrease the resistivity of the conductive traces.

Fully printed smart structures frequently have the printed conductors fully embedded in the structure. Embedding the conductive trace with PLA insulating traces was shown to significantly increase the resistivity (by approx. 50%) of the conductive trace.

Based on the findings of this research, the design and fabrication of future, fully-embedded, smart structures will be easier and more reliable.

## Figures and Tables

**Table 1:** Determined ranges for defined process parameters.

Process Parameter	Symbol	Range			Number of Values	Unit
		Min.	Max.	Step		
layer height	*h*	0.1	0.3	0.05	5	mm
trace spacing	*d*	0.25	0.65	0.1 (0.05)	6	mm
printing speed	*v*	10	40	5	7	mm/s
extrusion rate	$E_{r}$	90	130	10	5	%
nozzle temperature	$T_{\mathrm{nozzle}}$	100	200	20 (5)	9	${}^{\circ }C$
bed temperature	$T_{\mathrm{bed}}$	30	110	10	9	${}^{\circ }C$

**Table 2:** Default values of constant parameters for every varying parameter.

Researched Parameter	Constant Parameter Values						
	*h* [mm]	*d* [mm]	*v* [mm/s]	$E_{r}$ [%]	$T_{\mathrm{nozzle}}$ [${}^{\circ }C$]	$T_{\mathrm{bed}}$ [${}^{\circ }C$]	*w* [mm]
layer height	/	0.65	20	110	145	40	0.45
trace spacing	0.2	/	20	110	145	40	0.45
printing speed	0.2	0.55	/	110	145	40	0.45
extrusion rate	0.2	0.65	20	/	145	40	0.45
nozzle temperature	0.2	0.55	10	100	/	40	0.45
bed temperature	0.2	0.40	15	110	145	/	0.45

**Table 3:** Resistivity measurement results: average resistivity and standard deviation (std.) for every parameter value consisting of five identical conductive samples printed on shared PLA sample board.

Parameter	Value	Unit	Mean ρ [$10^{-3}$ Ω cm]	Std. ρ [$10^{-4}$ Ω cm]
layer height	0.10	mm	5.67	1.21
	0.15		4.68	0.92
	0.20		4.67	0.36
	0.25		4.88	0.55
	0.30		4.86	1.33
trace spacing	0.25	mm	10.34	4.60
	0.35		8.18	7.52
	0.45		6.10	3.28
	0.55		4.72	2.15
	0.60		4.13	0.26
	0.65		4.15	0.82
printing speed	10	mm/s	5.13	1.10
	15		4.71	1.00
	20		4.96	1.42
	25		5.18	1.81
	30		4.78	2.57
	35		4.83	1.82
	40		4.67	1.74
extrusion rate	90	%	4.24	0.87
	100		4.31	1.09
	110		4.50	1.08
	120		4.66	0.98
	130		4.69	0.99
$T_{\mathrm{nozzle}}$	100	${}^{\circ }C$	7.75	3.65
	120		5.31	1.88
	140		4.66	0.69
	145		4.37	0.45
	150		4.85	1.13
	155		5.12	1.63
	160		6.66	7.68
	180		22.00	4.19
	200		50.47	3.83
$T_{\mathrm{bed}}$	30	${}^{\circ }C$	6.04	1.47
	40		6.07	1.93
	50		6.91	2.11
	60		11.53	6.78
	70		10.06	3.95
	80		6.53	5.24
	90		7.06	5.54
	100		5.36	3.86
	110		4.90	2.07
PLA insulation	a)	/	3.85	0.64
	b)		4.40	1.33
	c)		4.31	2.03
	d)		6.25	3.39
	e)		6.55	1.41
heating	Fan OFF	/	6.95	5.56
	Fan ON		4.92	1.77
	1	min	4.16	3.24
	5		3.92	3.09
	15		5.75	4.37
